# Are emotional states based in the brain? A critique of affective brainocentrism from a physiological perspective

**DOI:** 10.1007/s10539-019-9699-6

**Published:** 2019-08-21

**Authors:** Giovanna Colombetti, Eder Zavala

**Affiliations:** 1 Department of Sociology, Philosophy, and Anthropology, University of Exeter, Exeter, EX4 4RJ UK; 2 Living Systems Institute, University of Exeter, Exeter, EX4 4QD UK; 3 EPSRC Centre for Predictive Modelling in Healthcare, University of Exeter, Exeter, EX4 4QD UK; 4 Wellcome Trust Centre for Biomedical Modelling and Analysis, University of Exeter, Exeter, EX4 4QD UK; 5 College of Engineering, Mathematics and Physical Sciences, University of Exeter, Exeter, EX4 4QD UK

**Keywords:** Emotions, Affective science, Brainocentrism, Stress, HPA axis, Gut microbiota

## Abstract

We call *affective brainocentrism* the tendency to privilege the brain over other parts of the organism when defining or explaining emotions. We distinguish two versions of this tendency. According to *brain-sufficient*, emotional states are entirely realized by brain processes. According to *brain-master*, emotional states are realized by both brain and bodily processes, but the latter are entirely driven by the brain: the brain is the master regulator of bodily processes. We argue that both these claims are problematic, and we draw on physiological accounts of stress to make our main case. These accounts illustrate the existence of complex interactions between the brain and endocrine systems, the immune system, the enteric nervous system, and even gut microbiota. We argue that, because of these complex brain–body interactions, the brain cannot be isolated and identified as the basis of stress. We also mention recent evidence suggesting that complex brain–body interactions characterize the physiology of depression and anxiety. Finally, we call for an alternative dynamical, systemic, and embodied approach to the study of the physiology of emotions that does not privilege the brain, but rather aims at understanding how mutually regulating brain and bodily processes jointly realize a variety of emotional states.

## Introduction

A long-debated question in affective science and emotion theory concerns the relationship between emotions and bodily changes or processes (in this field, “bodily changes” and “bodily processes” are used interchangeably to refer to physiological processes that occur specifically *outside* the brain). There are many theories and definitions of emotions, and they vary in how they conceive of the role of bodily changes. According to some theories, emotions are essentially cognitive states, while bodily changes are mere contingent concomitants. According to others, bodily changes are necessary constituents of emotions. Yet other theories regard emotions as necessarily involving both cognitive and bodily elements (see Scarantino and de Sousa 2018 for an overview of different theories and definitions of emotions in philosophy and affective science). Arguably, the prevailing approach in affective science today is the so-called “pattern” or “componential” view, according to which emotions are constituted by various components that co-vary with one another. These components typically include cognitive appraisals, facial expressions, action tendencies, autonomic or physiological arousal, and sometimes also feelings (Newen et al. 2015; Scherer 2009).

Despite this trend toward regarding bodily changes as important components of emotions, it is often claimed that the brain is the basis or substrate of emotions; or, equivalently, that emotions are based in the brain (e.g., LeDoux 1996, p. 8; Davidson et al. 2003; Lindquist et al. 2012; Pessoa 2017). What exactly this means is generally left unexplained. It is clear, however, that this claim goes together with the tendency to *privilege the brain over other physiological processes when defining or explaining emotions*. We call this tendency *affective brainocentrism* and we distinguish two versions of it more precisely in the next section. Unsurprisingly, perhaps, this tendency is often found in affective neuroscience; yet it also surfaces in philosophy, psychology, and even physiology.

We think that affective brainocentrism is problematic and our goal in this paper is to explain why. Our main claim is that interactions between brain and body (understood broadly to encompass commensal microorganisms) are too complex to warrant isolating and identifying brain processes as the basis of emotional states. Relatedly, bodily processes are not mere contingent accompaniments, or outputs, of such states. They are proper realizers of emotional states, together with brain processes. In other words, emotional states are physically realized by mutually regulating brain and bodily processes.

We support this view mainly by drawing on physiological accounts of stress. Different authors sometimes use the term “stress” to refer to quite different things. We use it to refer to an emotional state or condition that individuals can be in, and we take physiological accounts of stress to illustrate the physical processes that realize this emotional condition. These processes are quite well understood by now, especially compared to those of other emotional states. Importantly for our discussion, they involve complex reciprocal influences among brain and bodily systems—endocrine systems in particular, but also the immune system, the enteric nervous system, and even the gut microbiota. The physiology of stress thus presents a powerful case against affective brainocentrism. We also mention recent evidence suggesting that reciprocal brain–body influences characterize the physiology of other emotional conditions too, such as depression and anxiety. Although these influences are still only partially understood, the available evidence challenges the view that those emotional conditions are based in the brain. In the last section, we conclude by explicitly calling for a dynamical, systemic, and embodied approach to the study of the physical realizers of emotional states. We argue that this approach needs to be adopted not only in the case of stress, depression, and anxiety, but other emotional states too. We also briefly reflect on why it may be that recognition of the complex interconnectedness of brain and bodily processes, widespread in biology, still co-exists with brainocentric claims.

## Two versions of affective brainocentrism

Affective brainocentrism is most apparent in the claim that emotions are brain states. According to a recent working definition by two neuroscientists, “emotions are *internal brain states* that *cause* observable external changes in behaviour; observable internal physiological changes in the state of the body; changes in other mental states; and, under some conditions and in some species, changes in what we are consciously aware of” (Adolphs and Anderson 2018, p. 30; italics in original). In the course of the book they reiterate that emotions are “central states”, which they take to be synonymous with “internal brain states”. Their approach is a good illustration of what we call *brain-sufficient*: the brainocentric claim that the brain is sufficient for affective states. Philosophers have made this claim too. Thagard, for example, writes: “[b]asic emotions like happiness, sadness, fear, anger, disgust, and surprise can all be understood as brain processes, as can more complex social emotions such as shame, guilt, contempt, envy, pride, and gratitude” (2010, p. 94). The view that the brain entirely realizes an individual’s mental states is widespread among philosophical materialistic accounts of the mind. Wilson (2001) calls it “the standard view of realization”. According to it, “physical states of individuals—more particularly, of the central nervous system of individuals—are the physical realizations of an individual’s mental states, and these realizers are metaphysically sufficient for the presence of the states they realize” (Wilson 2001, p. 2). In the case of emotional states, this standard view is also manifest in influential works (both academic and popular-scientific) that call the brain “emotional”, “affective”, “happy”, and so on (see Table 1).^1^ In line with *brain-sufficient*, qualifying the brain as emotional, affective, etc. implies that properties of the brain alone determine the presence of emotional states. Methodologically, this view entails that, to explain the properties of an individual’s emotional state, one just needs to look at brain activity.

**Table 1:** A selection of literature representing the brainocentric perspective (*brain-sufficient*)

Author, date	Title (book or article)
Simonov (1986)	*The Emotional Brain*
LeDoux (1996)	*The Emotional Brain*
Dalgleish (2004)	The emotional brain
Wehrenberg and Prinz (2007)	*The Anxious Brain*
Davidson and Begley (2012)	*The Emotional Life of your Brain*
Pessoa (2013)	*The Cognitive–Emotional Brain*
Gasque (2016)	Seven glimpses into the emotional brain
Pessoa (2017)	A network model of the emotional brain
Burnett (2018)	*The Happy Brain*

Affective brainocentrism also comes in a subtler form, which we call *brain-master*. This is the view that the brain initiates emotions, and drives or controls their unfolding. LeDoux, for example, writes that “emotions come from brains” (1996, p. 13) and that the brain is the system that “generates” affective responses (1996, p. 18; see also LeDoux 2000). Panksepp (1998) repeatedly claims that the brain “generates”, “creates”, “produces”, as well as “controls” emotional behaviours and feelings (see for example the initial section on “Conceptual Background”; see also Panksepp 2005; Panksepp et al. 2017). Lindquist et al. (2012, p. 121; 172) regard understanding how the brain “creates” emotions as a long-standing and important goal of affective science. Even Adolphs and Anderson, who, as we saw, define emotions as brain states, at times also say that emotions “are produced by the brain” (2018, p. 12). This version of brainocentrism is subtler than the previous one because it does not identify the physical realizers of emotions with brain processes only. According to it, emotions are not just “embrained” but *embodied*, in the sense that they are physically realized not just by brain processes but also bodily ones. Nevertheless, this account is still brainocentric, as it privileges the brain by characterizing it as the generator and controller of (the rest of) emotional states. This account also regards bodily changes occurring during emotions as mere products or outputs of brain activity.

Importantly, affective brainocentrism is not just the claim that brain processes contribute to bringing about emotions. There is ample evidence supporting this claim, and we do not take issue with it here. In humans, for example, evidence from brain lesions has shown that damage to specific parts of the brain (such as the amygdala and the insula) results in impaired emotional behaviour or blunted feelings for specific emotions (fear and disgust, respectively) (Damasio et al. 2003; Adolphs and Anderson 2018, chapter 8). This evidence, however, supports at most the claim that those brain areas are necessary for (at least some) emotions. It does not support the stronger brainocentric claim that those brain areas are also sufficient. Nor does it support the brainocentric claim that those areas have a privileged role in causing affective states (i.e., by creating or driving them) compared to bodily processes—for the simple reason that brain lesion studies can only tell us something about the causal contribution of processes occurring in the brain. Likewise for those few electrical-stimulation studies in humans that found that stimulating certain areas of the brain elicited specific emotional feelings or behaviours, such as deep sadness, mirth and laughter, or disgust and nausea-related sensations (Adolphs and Anderson 2018, chapter 8, pp. 229–230). Another important source of evidence that brain activity plays a role in emotional states comes from animal studies that involve the manipulation of specific brain areas and the observation of subsequent behavioural changes. In particular, much is known today about brain processes involved in fear conditioning, largely thanks to the development of technologies that allow manipulating individual neurons. For example, there is evidence that activating neurons in specific nuclei of the rodent amygdala produces freezing (Adolphs and Anderson 2018, chapter 6). This type of evidence, like the one from stimulation studies in humans, contributes to showing that specific neurons causally influence behaviours typically associated with certain emotions. Once again, however, it does not support (either version of) affective brainocentrism—because, once again, it cannot rule out a possible role of bodily processes too.

## Challenging affective brainocentrism: the case of stress

We now turn to recent physiological accounts of stress to challenge both versions of affective brainocentrism. In this section and the next we argue that neither *brain-sufficient* nor *brain-master* can accommodate these accounts.

To clarify, we use “stress” to refer to an emotional state or condition that one can be in. This is the condition of “being stressed”, just as one can be in the emotional condition of being happy, scared, depressed, anxious, and so on. Some authors use “stress” differently, to refer to life-threatening or challenging circumstances that put pressure (i.e., stress) on the organism. We prefer to call these “stressors” (as they are also often called). Because stress occurs in response to stressors, we also sometimes talk of the “stress response” (just as happiness, fear, etc., are often referred to as “emotional responses” to indicate that they are responses to events). Scientific accounts also use this term frequently to describe physiological processes occurring during stress.

We take stress to be realized physically (like any other emotion and mental state more generally), and the question we are interested in here is what the physical realizers of stress are. We thus look at physiological accounts of stress, which describe these physical realizers. Physiological accounts are often contrasted with psychological ones, which aim primarily at identifying the cognitive processes that lead individuals to become stressed. Psychological accounts importantly emphasise individual differences in how individuals respond to the same circumstances, including the fact that some individuals but not others find certain circumstances stressful. These accounts, however, have not much to say about the physical processes realizing stress.^2^

Finally, in what follows we are not concerned specifically with *feelings*. Although feelings are part of the folk-psychological understanding of stress, we do not regard them as necessary for being stressed (in line with arguments for the existence of unconscious or unfelt emotions; e.g., Scarantino 2010). Our concern is with the physical realizers that constitute the (not necessarily conscious) condition of being stressed, and our aim is to emphasize that these realizers are not all in the brain.

### The standard physiological account

Consider first what we may call “the standard physiological account of stress”, as is typically presented in handbooks and review papers (for a general introduction, see Widmaier et al. 2014, chapter 11 section D; for a more detailed description, see Everly and Lating 2013). This account divides the stress response into two branches, both involving the hypothalamic–pituitary–adrenal (HPA) axis. This is constituted by the hypothalamus (located in the brain), the pituitary gland (located outside the brain, in a bony hollow behind the bridge of the nose just below the hypothalamus), and the adrenal glands (located on top of each kidney). One branch involves increased activity of the sympathetic nervous system, leading to increased secretion of epinephrine and norepinephrine from sympathetic neuron terminals and from the innervated adrenal medulla (the innermost part of the adrenal glands). This branch is responsible for the so-called “fight or flight response”, already studied by Cannon (1932). In the other branch, stressors stimulate the hypothalamus to release corticotropin-releasing hormone (CRH) and arginine vasopressin (AVP), which reach the pituitary through a blood portal system and via axons, respectively. In the pituitary, these hormones stimulate the secretion of adrenocorticotropic hormone (ACTH), which travels through the bloodstream to the adrenal cortex (which encloses the medulla) and stimulates the synthesis and secretion of glucocorticoids (e.g., cortisol in humans). Alongside epinephrine and norepinephrine, glucocorticoids are often called “stress hormones”.

**Fig. 1:**
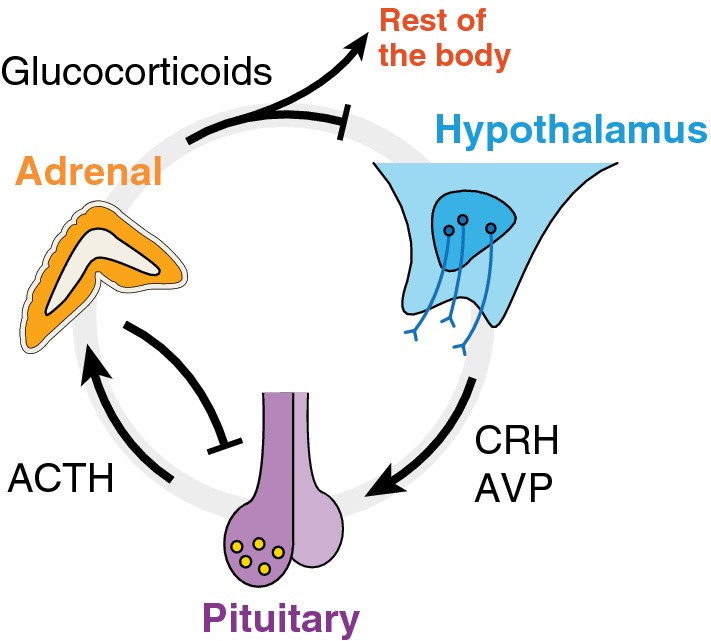
Dynamic regulation within the HPA axis. Positive (negative) interactions are indicated by arrows (edges). Once secreted, glucocorticoids inhibit their own synthesis via negative feedback loops with the pituitary and the hypothalamus

Glucocorticoids are rapidly secreted (Spiga et al. 2017) to prepare the body to deal with stressors (for example, they trigger anti-inflammatory and immunosuppressive effects, and affect glucose expenditure). In addition, and importantly for our argument, they also regulate their own synthesis by inhibiting the secretory activity of the pituitary and hypothalamus, which downregulates further release of ACTH and, consequently, reduces glucocorticoid synthesis (Fig. 1). In healthy organisms, this is the homeostatic (or rather homeodynamic) negative feedback process that prevents prolonged exposure to high levels of circulating glucocorticoids.

### Implications for brain-sufficient

Before considering further physiological details, note that the account described so far is already evidently at odds with *brain-sufficient*. It characterizes stress as realized not by brain activity only, but by neuroendocrine processes that span brain *and* body. Relatedly, the standard physiological account already entails that looking at the brain only cannot provide an adequate explanation of how organisms respond to stressors.

A corollary of this point is that the standard physiological account is also at odds with the view that bodily changes are mere outputs of stress. This view can be found for example in Panksepp (1998, p. 119). He acknowledges that various bodily responses (immune, visceral, enteric) “are recruited during stress”, and that they influence the brain through nervous and humoral paths. Nevertheless, he still characterizes stress as “centrally integrated” in limbic areas of the brain, and bodily processes as the “output” of activity in these areas. He explicitly claims that the task of describing the brain circuitry of stress has priority over the one of describing bodily effects (“the more basic and crucial task is to explore the central brain systems that mediate emotionality”). Yet all he says in support of this privileging of the brain is that “the brain itself contains many similar neural systems” to those of the peripheral nervous system—seemingly implying that studying these neural systems in the brain is sufficient for studying stress. Clearly, however, this similarity entails neither that stress is “centrally integrated” in the brain nor that looking at brain processes “is the more basic and crucial task”. Moreover, as we saw, the physiology of stress involves not just neural processes but also endocrine ones that are not “contained” in the brain, and that an exclusive focus on brain activity is bound to miss.

The standard physiological account fits naturally with an embodied view of the condition of being stressed, according to which both brain and bodily processes are physical realizers. Consider an analogy with digestion. Handbook physiological accounts of the digestive process characterize it as starting already in the mouth, where enzymes contained in the saliva begin to break food down. Food then reaches the stomach, which secretes various gastric juices. Simultaneously, other organs (e.g., liver, pancreas, gallbladder) secrete enzymes that facilitate the breakdown of complex molecules into glucose and regulate glucose homeostasis (Widmaier et al. 2014, chapter 15). Given this account, to claim that the digestive process is entirely realized by processes taking place in the stomach would be unwarrantedly narrow and misleading, because it would leave out organs and activities that are regarded as constitutive parts of the physiology of digestion. The common understanding of the physiology of digestion is that it includes not just processes occurring in the stomach but also in the mouth, liver, pancreas, gallbladder, as well as in organs of the gastrointestinal tract that contribute further to breaking food down into nutrients (ileum, duodenum, small bowel, and colon). We claim an analogous case for stress. The common understanding of stress, given what is known about its physiology, is that it is realized by processes occurring in both brain and body. To regard stress as taking place in the brain only would be unwarrantedly narrow and misleading, given what we know about the influence of bodily processes on the organism’s response to stressors and on brain activity itself.

One might remark, at this point, that it is not clear that activation of the HPA axis is relevant for criticizing brainocentrism, because historically this axis initially comprised only the pituitary and the adrenal glands, while the hypothalamus was added later on. Indeed, Selye’s (1936) first description of the general adaptation syndrome only mentioned changes in the body. Only later did he mention the hypothalamus as a possible initiator of the syndrome (Selye 1950); and, even then, he still described the syndrome entirely in terms of bodily changes, focusing on endocrine processes. This historical consideration, however, does not undermine our argument. Arguably it reinforces it, as it may be used to emphasize that bodily processes have long been regarded as parts of the physical realization of stress. It is certainly an interesting question why, from an initial focus on the body, accounts of this physical realization have shifted toward a more brain-based perspective—such as Panksepp’s (1998) view mentioned above, but also, as we are about to see, in recent physiological accounts.^3^

### Implications for brain-master, and further physiological details

Having argued that it is implausible to maintain *brain-sufficient* in the case of stress, we now turn to *brain-master*. Is it plausible to regard the brain as the master regulator that initiates, and drives or controls, the organism’s response to stressors? *Brain-master* is arguably the prevailing view in contemporary physiological accounts of stress. These often describe the brain as containing structures that evaluate the significance of a situation as threatening, and structures that initiate and control a series of responses in the body that allow the organism to cope with the situation (e.g., McEwen 2006, 2007; Everly and Lating 2013). This view is similar to cognitive-componential approaches to emotion, according to which cognitive components of emotions elicit and drive the bodily components (e.g., Scherer 2009). McEwen, for example, explicitly claims that the brain “is the key organ of stress” (2006, p. 367) and “the master regulator of the neuroendocrine, autonomic, and immune systems” (2006, p. 371).

Interestingly, *brain-master* is often endorsed along with the acknowledgment of the existence of bidirectional influences between brain and body. The same scientists who regard the brain as initiating and driving the physiological stress response also talk of the “systemic circulation” (Everly and Lating 2013, p. 43) or the “nonlinearity” (McEwen 2006, p. 370, 2007, p. 881) that characterizes brain–body interactions during this response. This acknowledgement, however, is in tension with *brain-master*, because it entails not only that the brain influences the stress response as it unfolds, but also that the brain’s influence on this response is itself regulated by bodily processes. Importantly, the stress response involves not only short-term bodily influences on the brain, but also long-term ones. In chronic stress (induced by prolonged and repeated exposure to stressors), stress hormones have been found to induce structural changes in the hippocampus, amygdala, and prefrontal cortex, with related alterations in cognitive and behavioural functions (McEwen 2007). It is difficult, we maintain, to uphold *brain-master* in the face of this evidence.

One might retort that it is possible to admit the existence of influences from the body to the brain, while also maintaining that the brain is still what drives the physiological and behavioral responses characteristic of being stressed. A tacit widespread assumption here appears to be that physiological processes are organized hierarchically, with the brain sending out instructions to the body and then modifying them in light of incoming signals from it. Indeed, the brain is often said to “control” bodily processes, whereas the latter are said merely to “modulate” the brain (e.g., Davidson et al. 2003, p. 4, write that “while the central nervous system obviously represents and controls the autonomic nervous system, there is also important feedback from the ANS to the central nervous system, that serves to modulate central function”; see also McEwen 2006, 2007). “Modulation” refers to a modification of an ongoing process that has been triggered by something else. There is, in other words, an *asymmetry* in the characterization of the causal powers of brain and body. The brain is taken to “hold the reins” of stress by driving its unfolding; the body is granted causal power, but only a “modulatory” one (rather than a fully “controlling” one), revealing the assumption that its influence is somehow less important than that of the brain. Note, though, that this asymmetry is typically just stated rather than justified on the basis of evidence and arguments. If we keep to the available evidence, all it shows is that there are reciprocal influences involved in the physiology of stress, such that without certain brain processes some bodily processes do not occur—and, importantly, vice versa.^4^

**Fig. 2:**
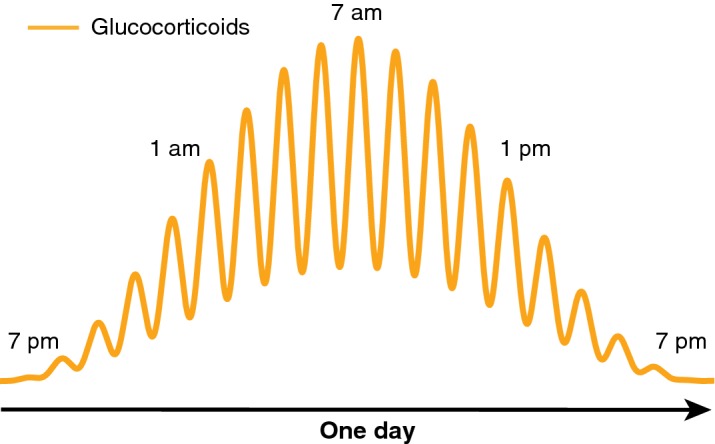
Schematics of circadian (24 h) and ultradian ( ~ 1 h) oscillations in plasma concentrations of glucocorticoids

Moreover, there is additional evidence about the physiology of the HPA axis that further undermines *brain-master*. It has been known since the 1950s that plasma concentrations of glucocorticoids fluctuate with a 24-h periodicity (circadian variation), with maximum values observed just before awakening (for an overview, see Kalsbeek et al. 2012). More recently, it has become clear that the concentrations of these hormones increase and decrease also with a near-hourly rhythm (ultradian oscillations) (Russell et al. 2015). The combination of these fluctuations gives rise to the pattern represented in Fig. 2.

The significance of this oscillatory pattern is the subject of much research, and there is evidence showing that its chronic disruption is associated with both physiological and behavioural disorders (Lightman and Conway-Campbell 2010; Russell et al. 2015; Spiga et al. 2015). Importantly, whereas the hypothalamus had been hypothesised to be the source of ultradian oscillations by acting as a pulse generator (Mershon et al. 1992), later theoretical models predicted that feedback interactions between the pituitary and adrenal glands would suffice to generate ultradian pulsatility (Walker et al. 2010). Subsequent animal studies have confirmed this model by showing that these oscillations persist even when pulsatile signals from the brain are blocked (Waite et al. 2012). In other words, the ultradian rhythm is generated and regulated by pituitary–adrenal interactions, independent of the hypothalamus.

Particularly significant for our argument is the finding that the magnitude of the organism’s response to a stressor depends on whether the stressor occurs during either the rising or falling phase of the ultradian glucocorticoid oscillation. Studies in rats conducted in the late 1990s already showed that the magnitude of the organism’s response to an acute noise stressor applied during the rising phase is enhanced, relative to the magnitude of the response to the same stressor applied during the falling phase (Windle et al. 1998a, b, 2001; see also Rankin et al. 2012). Later studies in male rats indicated the existence of a correlation between ultradian glucocorticoid rhythms and the propensity to behave aggressively (Haller et al. 2000, 2001). In these studies, not only the circulating levels of glucocorticoids affected the patterns of aggression toward an intruder (social stressor), but rats were significantly more aggressive when confronting the intruder in the rising phase of their ultradian glucocorticoid fluctuation, compared to counterparts in the decreasing phase.^5^

These findings further undermine *brain-master*, in that they demonstrate that the organism’s response to stressors is at least partly controlled by oscillating bodily processes and by the direction of this oscillation—with research indicating, importantly, that this oscillation is independent of the brain. They also further undermine *brain-sufficient*, because they provide additional evidence that factors outside the brain (and not under its influence) are indispensable for explaining the occurrence and unfolding of the stress response, and moreover for explaining how the brain reacts during stress. In the face of this evidence, upholding *brain-sufficient *for the case of stress is still a possible metaphysical option, but one increasingly at odds with current scientific explanatory practices and findings. We think that the latter ought to have a say in establishing what processes count as physical realizers of emotional states—including displacing the view that stress is a brain process, when it is evident that mutual influences between brain and body make both explanatorily indispensable.

In sum, physiological evidence from neuroendocrinology undermines the view that stress is based in the brain, in both the *brain-sufficient* and *brain-master* sense. There is thus at least one emotional state that is not brain-based. In the next section, we challenge affective brainocentrism further. First, we argue that the physiological realizers of stress can be seen as extending even beyond the neuroendocrine processes described in this section. Second, we present some evidence suggesting that other emotional states, such as depression and anxiety, are not brain-centred either.

## The role of other physiological systems in stress and other emotional conditions

It would not be accurate to conclude that the physiological realizers of stress are limited to the neuroendocrine processes described in the previous section. Scientific research in the last 15 years has begun to show just how more complex the physiology of stress is, and how difficult it is to draw fixed uncontroversial boundaries around its physical realizers. This research challenges brainocentrism further. It has now become clear that the processes described so far are deeply interrelated with the gastrointestinal tract (Konturek et al. 2011). Stressful situations influence gut physiology at many levels, e.g., alter gastrointestinal motility and secretion, increase intestinal permeability, and modify intestinal microbiota (the many different bacteria and other microorganisms that live in the gut). They also induce the release of various neurotransmitters (from both the central and enteric nervous system) and proinflammatory cytokines (from the immune system), both of which influence gut physiology. Vice versa, a growing body of evidence shows that the condition of the gut affects the organism’s response to stressors. Gut–brain connections began to be investigated already in the first half of the twentieth century, although current researchers usually cite Sudo et al. (2004) as the starting point of contemporary work on the so-called “microbiota–gut–brain axis” (see Hooks et al. 2019). Sudo et al. showed that germ-free mice (mice raised in the absence of microorganisms) displayed disproportionally elevated levels of glucocorticoids in stressful situations, and that recolonizing the mice with individual strains of microbes selectively influenced the activity of the HPA axis. Since then, several other studies have looked at the relation between stress and the microbiota–gut–brain axis in rodents. Overall, they support the view that gut microbiota are influenced by, and influence, the stress response (Sarkar et al. 2018)—even though not much is known about the specific causal pathways and mechanisms of these interconnections (Hooks et al. 2019). In humans, the effects of bacteria on stress have been studied mainly by looking at the effects of probiotics—defined as living microorganisms that, when ingested, contribute to bringing about some health effect (possibly by altering the gut microbiota). Some studies have found that consuming probiotics (compared to a placebo) reduces cortisol or prevents its increase in stressful circumstances, among other effects (e.g., Messaoudi et al. 2011; Kato-Kataoka et al. 2016).

In addition, interactions between brain, gastrointestinal tract, and gut microbiota appear to be mediated by other bodily systems—such as the enteric nervous system and the immune system (Rhee et al. 2009; Konturek et al. 2011; Lyte et al. 2011). If it is the case that exposure to stressors affects all these systems and their relations, and vice versa, restricting the physiology of stress to neuroendocrine processes on the HPA axis is arguably too narrow. In fact, the very enterprise of looking for *the* physical realizers of stress as if these had clear, fixed boundaries, is starting to appear incomplete at best, and misguided at worst. It may well be wisest, then, to give up trying to identify a fixed “basis” of stress altogether.

These considerations can be extended to argue that the brain is not the basis of other emotional conditions either, such as depression and anxiety. There is evidence of reciprocal links between chronically elevated glucocorticoids, the brain, and the immune system in depression (see Maier and Watkins 1998; Dantzer et al. 2008; Slavich and Irwin 2014). During inflammation, the immune system produces pro-inflammatory cytokines that influence the brain, and the brain in turn sends signals to inhibit the inflammatory process. Interestingly, pro-inflammatory cytokines appear to have at least some effects on depressive symptoms (such as low mood, anhedonia, fatigue, and socio-behavioural withdrawal), and a few studies have shown that anti-inflammatory agents alleviate these symptoms (reviewed in Slavich and Irwin 2014). Claims have been made that gut bacteria, too, may have an influence on depression and anxiety in humans, as well as on depression-like and anxiety-like behaviour in rodents (reviewed in Cryan and Dinan 2012; Foster and Neufeld 2013; Sarkar et al. 2018). For example, faecal transplants from mice displaying high anxiety-like behaviour to “non-anxious” mice produce an anxious-like phenotype (i.e., mice displaying reduced exploratory behaviour), and vice versa for transplants from mice displaying low anxiety-like behaviour to “anxious” mice (reviewed in Sarkar et al. 2018). Administration of a specific strand of bacteria in mice has been shown to alter anxiety-like and depression-like symptoms via influences on GABA receptor expression (Bravo et al. 2011). Tryptophan-metabolizing gut microbiota contribute to changes in the amount of serotonin in the gastrointestinal epithelium (where most of the body’s serotonin is produced), and serotonin is known to be one of the key neurotransmitters altered in depression (Jenkins et al. 2016). Recent reviews acknowledge a possible role for probiotics in alleviating depressive symptoms in humans, although they also emphasize the need for further research and double-blind randomized control trials (Wallace and Milev 2017; Ng et al. 2018; Park et al. 2018).

Importantly, this research has not gone unchallenged. A recent critical analysis (Hooks et al. 2019) highlights various methodological and conceptual shortcomings, including the tendency to draw simplistic conclusions and make bold claims about how the gut microbiota, or specific bacterial strands, influence (even “control” or “manipulate”) brain, behaviour, and mental states (see also Taylor 2019 for similar concerns about microbiota–gut–brain research applied to mental health; for broader criticisms of causal claims about microbiota, see Hanage 2014). Nevertheless, it is well established by now that there are various forms of reciprocal influences between brain and body (including gut microbiota). Additionally, there is increasing evidence that complexly interrelated processes straddling brain and body influence, and are influenced by, emotional conditions such as stress, depression, and anxiety. Even though the causal pathways linking specific physiological changes to aspects of affective behaviour and experience remain poorly understood, the implication is that focusing solely on the brain can provide only a very limited account of the physical realization of those conditions.

## Concluding remarks

In sum, we have argued that affective brainocentrism is problematic by showing that it is not compatible with current physiological accounts of at least some emotional states or conditions. Evidence from physiology shows that when an individual is stressed, depressed, or anxious, these conditions are sustained through complex interactions between brain and bodily processes. This complex interactivity undermines both the claim that the brain is the sole realizer of those conditions, and that it is their locus of control or master regulator. It also undermines the methodological assumption that studying the brain only can provide adequate and complete explanations of those emotional conditions (as in Panksepp’s claim that “the more basic and crucial task is to explore the central brain systems that mediate emotionality”).

What then follows from our argument? In this concluding section we highlight a number of varied points. First, in our view affective brainocentrism needs to be abandoned. Research into the physiology of emotional states should proceed by recognising from the start the deep integration of brain processes with the rest of the organism (including commensal microorganisms). Proper recognition of this biological fact should lead to taking the body more seriously than is often the case in affective science, where bodily changes are usually assumed to be secondary or ancillary to the brain (either as mere side-effects of brain-based emotions; or, at most, as modulators of brain-driven emotions). It should lead instead to regarding brain and body as jointly contributing, through mutually regulatory processes, to the occurrence and temporal unfolding of emotional states.

This proposal amounts, in effect, to an invitation to adopt a dynamical, systemic, and embodied approach to the study of the physiology of emotional states. Taking such an approach means looking at how physiological processes in both brain and body unfold and change over time, and influence one another; and at how their mutual influences over time make a difference to the individual’s emotional condition. The study of the physiology of stress illustrates this approach particularly well. In our opinion, it provides the most detailed account available of brain–body influences at different timescales in the case of an emotional condition. Accounts of the physiology of depression and anxiety that emphasize the influence of bodily processes, such as the immune system and the gut microbiota, are also relevant, although comparatively less detailed. Tracing the specific contributions of various brain and bodily processes to those conditions is a complex task, and it may take a long time to achieve the same level of detail and understanding as in the case of stress. Nevertheless, we think those accounts are important—not least because they challenge and counterbalance the tendency, in psychiatry, to reduce mental disorders to brain disorders (for an illustration of this tendency see Vidal and Ortega 2017, chapter 3). At the same time, adopting a dynamical, systemic, and embodied approach to those conditions also involves refraining from shifting most or even all regulatory burden from brain processes to bodily ones. As we have seen, claims have been made that the gut microbiota drive, govern, or manipulate us (Hooks et al. 2019). These claims are just as misleading as saying that the brain controls us. In a systemic framework, control is rather best understood as a function of the whole system achieved through the mutually regulatory influences of its constituent processes.

Second, what about the many other emotional states studied in affective science, such as fear, anger, happiness, disgust, guilt, shame, and so on? Do our considerations in this paper apply to them as well? Or is it more plausible to regard them as based in the brain—in the sense of either *brain-sufficient*, or at least *brain-master*? One might claim that all we have shown is that a few *moods* and *mood disorders* are not based in the brain; and that we have not provided any argument against the view that (most) emotions are based in the brain. This objection would appeal to the widespread distinction between emotions and moods. How exactly to draw this distinction is controversial, but some criteria are relatively well agreed-upon (see Stephan 2017). Many philosophers in particular regard emotions as intentional, namely, as directed at specific objects or states of affairs (one is angry *with* someone, afraid *of* something, happy *about* a certain situation). On the other hand, they regard moods as objectless, i.e., as not directed at anything. When one is in a bad mood, the bad mood is not about anything; similarly when one is grumpy, elated, or energized. According to this criterion, stress, depression, and anxiety are moods rather than emotions, as they are arguably not directed at anything. Another criterion, more common in psychology, is that emotions are short-lived while moods are longer-lasting. Emotions are taken to last from a few seconds to a few minutes. Moods, on the other hand, are taken to last from several minutes to hours, days, or even longer (clinical mood disorders, for example, can endure for months to years).

In response, note first that not all affective scientists draw this distinction clearly or absolutely. Eliciting emotions in the laboratory is sometimes called “mood induction” and is achieved through methods (e.g., music, autobiographical memories) that arguably elicit generalized objectless moods, rather than emotions (see discussion in Fox 2008, chapter 2). Relatedly, many affective scientists, including neuroscientists, use the term “emotions” to refer to both shorter and longer states. When they say that the brain generates or controls “emotions”, often they mean both brief emotional episodes and longer-lasting conditions such as (what some call) moods. Second, importantly long-lasting states such as those we have discussed here *influence* shorter-lived emotional episodes (Stephan 2017). We know from everyday experience and clinical research that being stressed can make one more aggressive and likely to become angry. Anxiety typically comes with frequent episodes of fear and worry, and depression with guilt and hopelessness. A possible explanation of these influences is that moods and mood disorders, as realized by integrated brain–body processes, set up a physiological landscape that alters the arousal threshold for specific emotions. As Ekman (1994, p. 57) puts it, moods “lower the threshold for arousing the emotions, which occur most frequently during a particular mood. … In an irritable mood people construe the world around them in a way that permits, if not calls for, an angry response”. If this is correct, to regard moods as realized by integrated brain–body processes, and emotions as based in the brain, is to adopt a narrow and therefore arguably misleading perspective—one that does not sufficiently acknowledge the contribution that longer-lasting brain and bodily processes jointly make to the occurrence of short-lived episodes.

More generally, we do not think that the domain of emotional states can be strictly divided into those that are brain-based and those that are not. The brain, after all, is *always* deeply interconnected with the rest of the organism—not just when one is stressed, anxious, or depressed. We have mentioned evidence of specific brain–body interactions occurring during these conditions, but we do not want to suggest that brain and body are otherwise decoupled. brain–body integration is the norm, not the exception. Accordingly, it seems implausible to reject brainocentrism for some emotional states but not for others.

Third, one might wonder why brainocentrism persists in affective science, given that the integration of brain and body is old news in biology and neuroscientists are also well aware of it. It is indeed interesting that some researchers, as we have seen, make brainocentric claims even when they explicitly note that brain and body are complexly interrelated. Awareness of the deep interconnectedness of brain and body has not led to abandoning the assumption that the brain can somehow be unplugged from the rest of the organism and take on the role of sole realizer, or master regulator, of emotional states.

Why this happens, we suspect, has no straightforward explanation. One reason might be that, historically, affective science has had stronger methodological and theoretical links with cognitive science than with biology and physiology. That cognitive states are realized by brain processes is arguably the default position in mainstream cognitive science. This default position may just have been transferred to emotional states too. So strong is the grip of the default position, that the deep integration of brain and body, even though widely recognized, is not enough to dispel it. But why is brainocentrism so resilient? This is, we think, a complex socio-historical question. As Vidal and Ortega (2017) have recently argued, what they call “the ideology of the cerebral subject” is a deeply entrenched assumption in Western culture since at least the eighteenth century. By this phrase they refer to the assumption that “we are essentially our brains” (2017, p. 5). They define this assumption as an “ideology” because, they argue, it is not supported by empirical evidence. We think they have a point here—even though unfortunately they do not discuss the positive contribution that cognitive and affective neuroscience have made to the understanding of the causal roles of various brain processes to our mental life. As we noted before, empirical evidence does not support affective brainocentrism. Neither brain-lesion and brain-stimulation studies in humans, nor brain-manipulation studies in animals, can establish that the brain is the sole realizer or the master regulator of emotional states. This is because they do not provide evidence ruling out the possible role of other physiological processes. Absent this evidence, claims that emotions are based in the brain remain unwarranted and thus open to the charge of being ideological.

Finally, what about physical processes that occur in the environment outside the organism, including non-biological ones? Should we regard them as physical realizers of emotional conditions too, at least in some cases? The systemic perspective we favour implies a positive answer to this question. If certain environmental processes are closely integrated with physiological processes, such that this integration contributes to maintaining a certain emotional condition, we see no principled reasons why the environmental processes in question should not also be considered part of the physical realization of that emotional condition. Some philosophers have in fact already made this point as part of recent debates on the so-called “extended mind thesis” (e.g., Stephan et al. 2014; Krueger 2014; Colombetti 2017). Emotional states are said to “extend” into the environment when their physical realization is not limited to brain and bodily processes, but includes parts of the environment (from hormonal patches to musical instruments) closely coupled to the organism.

Although we sympathize with this view, in this paper we have chosen to focus specifically on biological integration at the organism level (including microbiota) to highlight physiological findings that we think should be given more attention in affective science, as well as philosophy. Surprisingly, perhaps, philosophical arguments that emphasize the embodied nature of the mind hardly make any reference to the interrelation of the brain with the peripheral nervous system, the endocrine system, the gut microbiota, etc. (see for example papers in Shapiro 2014; Newen et al. 2018). This literature overlooks these biological details, possibly as a consequence of functionalism in philosophy of mind, which generally disregards how mental functions are materially implemented. We hope to have shown that, on the contrary, biological details *do* matter for deciding how to conceptualize the relation between emotional states and their physical realizers—and, ultimately, for dispelling assumptions about the ontological and causal primacy of the brain that are deeply entrenched in philosophy, cognitive and affective science, and well beyond.

## Footnotes

1 There is even a lab—Tali Sharot’s laboratory at University College London—called “The Affective Brain Lab”.

2 Physiological accounts of stress are traditionally traced to Hans Selye’s notion of the general adaptation syndrome (originally advanced in Selye 1936). Psychological accounts are the legacy of Richard Lazarus’s (1966) work on cognitive appraisal and coping processes. For the complex history of the notion and science of stress, see Cooper and Dewe (2004) and Jackson (2013).

3 Answering this question would be a complex historical task, partly because of the existence of several different influences on current physiological accounts of stress. As a recent brief historical account shows (Miller 2018), since the 1920s empirical findings at different times led to different claims about possible loci of control of the HPA axis. Moreover, social and ideological factors arguably often trump empirical evidence when it comes to brainocentric claims (see last section).

4 A similar situation prevails for genes, and whether they encode or control phenotypic development (see Oyama 2000). The privileged status accorded to genes in some explanations is very similar to the status accorded to brains in some explanations of emotional states.

5 In humans, low glucocorticoid levels are associated with a propensity to aggression, disruptive behaviour and antisocial personality (Virkkunen 1985; Vanyukov et al. 1993; McBurnett et al. 2000), but the causal links between these and glucocorticoid dynamics are yet to be determined. A recent study in humans has shown that administering the same dose of cortisol in different dynamic patterns influences the brain’s responsiveness to emotional stimulation, accuracy in recognition, and attention bias toward/away from emotional faces (Kalafatakis et al. 2018).

## References

[CR1] Adolphs R, Anderson DJ (2018). The neuroscience of emotion: a new synthesis.

[CR2] Bravo JA, Forsythe P, Chew MV, Escaravage E, Savignac HM, Dinan TG (2011). Ingestion of Lactobacillus strain regulates emotional behavior and central GABA receptor expression in a mouse via the vagus nerve. Proc Natl Acad Sci.

[CR3] Burnett D (2018). The happy brain: the science of where happiness comes from, and why.

[CR4] Cannon WB (1932). The wisdom of the body.

[CR5] Colombetti G (2017). Enactive affectivity, extended. Topoi.

[CR6] Cooper CL, Dewe P (2004). Stress: a brief history.

[CR7] Cryan JF, Dinan TJ (2012). Mind-altering microorganisms: the impact of the gut microbiota on brain and behaviour. Nat Rev Neurosci.

[CR8] Dalgleish T (2004). The emotional brain. Nat Rev Neurosci.

[CR9] Damasio AR, Adolphs R, Damasio H, Davidson RJ, Scherer KR, Goldsmith HH (2003). The contributions of the lesion method to the functional neuroanatomy of emotion. Handbook of affective sciences.

[CR10] Dantzer R, O’Connor JC, Freund GG, Johnson RW, Kelley KW (2008). From inflammation to sickness and depression: when the immune system subjugates the brain. Nat Rev Neurosci.

[CR11] Davidson RJ, Begley S (2012). The emotional life of your brain: how its unique patterns affect the way you think, feel, and live—and how you can change them.

[CR12] Davidson RJ, Scherer KR, Goldsmith HH, Davidson RJ, Scherer KR, Goldsmith HH (2003). Introduction: neuroscience. Handbook of affective sciences.

[CR13] Ekman P, Ekman P, Davidson RJ (1994). Moods, emotions, and traits. The nature of emotion: fundamental questions.

[CR14] Everly GS, Lating JM (2013). A clinical guide to the treatment of the human stress response.

[CR15] Foster JA, Neufeld KAM (2013). Gut–brain axis: how the microbiome influences anxiety and depression. Trends Neurosci.

[CR16] Fox E (2008). Emotion science: cognitive and neuroscientific approaches to understanding human emotions.

[CR17] Furness JB (2000). Types of neurons in the enteric nervous system. J Auton Nerv Syst.

[CR18] Gasque G (2016). Seven glimpses into the emotional brain. PLoS Biol.

[CR19] Griffiths PE (1997). What emotions really are: the problem of psychological categories.

[CR20] Haller J, Halasz J, Mikics É, Kruk MR, Makara GB (2000). Ultradian corticosterone rhythm and the propensity to behave aggressively in male rats: ultradian corticosterone rhythm and aggression. J Neuroendocrinol.

[CR21] Haller J, van de Schraaf J, Kruk MR (2001). Deviant forms of aggression in glucocorticoid hyporeactive rats: a model for “pathological” aggression?. J Neuroendocrinol.

[CR22] Hanage WP (2014). Microbiology: microbiome science needs a healthy dose of skepticism. Nature.

[CR23] Hooks KB, Konsman JP, O’Malley MA (2019). Microbiota–gut–brain research: a critical analysis. Behav Brain Sci.

[CR24] Jackson M (2013). The age of stress: science and the search for stability.

[CR25] Jenkins T, Nguyen J, Polglaze K, Bertrand P (2016). Influence of tryptophan and serotonin on mood and cognition with a possible role of the gut–brain axis. Nutrients.

[CR26] Kalafatakis K, Russell GM, Harmer CJ, Munafo MR, Marchant N, Wilson A (2018). Ultradian rhythmicity of plasma cortisol is necessary for normal emotional and cognitive responses in man. Proc Natl Acad Sci.

[CR27] Kalsbeek A, van der Spek R, Lei J, Endert E, Buijs RM, Fliers E (2012). Circadian rhythms in the hypothalamo–pituitary–adrenal (HPA) axis. Mol Cell Endocrinol.

[CR28] Kato-Kataoka A, Nishida K, Takada M, Kawai M, Kikuchi-Hayakawa H, Suda K (2016). Fermented milk containing *Lactobacillus casei* strain Shirota preserves the diversity of the gut microbiota and relieves abdominal dysfunction in healthy medical students exposed to academic stress. Appl Environ Microbiol.

[CR29] Konturek PC, Brzozowski T, Konturek SJ (2011). Stress and the gut: pathophysiology, clinical consequences, diagnostic approach and treatment options. J Physiol Pharmacol.

[CR30] Krueger J (2014). Varieties of extended emotions. Phenomenol Cogn Sci.

[CR31] Lazarus RS (1966). Psychological stress and the coping process.

[CR32] LeDoux JE (1996). The emotional brain: the mysterious underpinnings of emotional life.

[CR33] LeDoux JE (2000). Emotion circuits in the brain. Ann Rev Neurosci.

[CR34] Lightman S, Conway-Campbell BL (2010). The crucial role of pulsatile activity of the HPA axis for continuous dynamic equilibration. Nat Rev Neurosci.

[CR35] Lindquist KA, Wager TD, Kober H, Bliss-Moreau E, Barrett LF (2012). The brain basis of emotion: a meta-analytic review. Behav Brain Sci.

[CR36] Lyte M, Vulchanova L, Brown DR (2011). Stress at the intestinal surface: catecholamines and mucosa–bacteria interactions. Cell Tissue Res.

[CR37] Maier SF, Watkins LR (1998). Cytokines for psychologists: implications of bidirectional immune-to-brain communication for understanding behavior, mood, and cognition. Psychol Rev.

[CR38] McBurnett K, Lahey BB, Rathouz PJ, Loeber R (2000). Low salivary cortisol and persistent aggression in boys referred for disruptive behavior. Arch Gen Psychiatry.

[CR39] McEwen BS (2006). Protective and damaging effects of stress mediators: central role of the brain. Dialogues Clin Neurosci.

[CR40] McEwen BS (2007). Physiology and neurobiology of stress and adaptation: central role of the brain. Physiol Rev.

[CR41] Mershon JL, Sehlhorst CS, Rebar RW, Liu JH (1992). Evidence of a corticotropin-releasing hormone pulse generator in the macaque hypothalamus. Endocrinol.

[CR42] Messaoudi M, Lalonde R, Violle N, Javelot H, Desor D, Nejdi A (2011). Assessment of psychotropic-like properties of a probiotic formulation (*Lactobacillus helveticus* R0052 and *Bifidobacterium longum* R0175) in rats and human subjects. Br J Nutr.

[CR43] Miller WL (2018). The hypothalamic–pituitary–adrenal axis: a brief history. Horm Res Paediatr.

[CR44] Newen A, Welpinghus A, Juckel G (2015). Emotion recognition as pattern recognition: the relevance of perception. Mind Lang.

[CR45] Newen A, Bruin LD, Gallagher S (2018). The Oxford handbook of 4E cognition.

[CR46] Ng QX, Peters C, Ho CYX, Lim DY, Yeo WS (2018). A meta-analysis of the use of probiotics to alleviate depressive symptoms. J Affect Disord.

[CR47] Oyama S (2000). Causal democracy and causal contributions in developmental systems theory. Philos Sci.

[CR48] Panksepp J (1998). Affective neuroscience: the foundations of human and animal emotions.

[CR49] Panksepp J (2005). On the embodied neural nature of core emotional affects. J Conscious Stud.

[CR50] Panksepp J, Lane RD, Solms M, Smith R (2017). Reconciling cognitive and affective neuroscience perspectives on the brain basis of emotional experience. Neurosci Biobehav Rev.

[CR51] Park C, Brietzke E, Rosenblat JD, Musial N, Zuckerman H, Ragguett R-M (2018). Probiotics for the treatment of depressive symptoms: an anti-inflammatory mechanism?. Brain Behav Immun.

[CR52] Pessoa L (2013). The cognitive–emotional brain: from interactions to integration.

[CR53] Pessoa L (2017). A network model of the emotional brain. Trends Cogn Sci.

[CR54] Rankin J, Walker JJ, Windle R, Lightman SL, Terry JR (2012). Characterizing dynamic interactions between ultradian glucocorticoid rhythmicity and acute stress using the phase response curve. PLoS ONE.

[CR55] Rhee SH, Pothoulakis C, Mayer EA (2009). Principles and clinical implications of the brain–gut–enteric microbiota axis. Nat Rev Gastroenterol Hepatol.

[CR56] Russell GM, Kalafatakis K, Lightman SL (2015). The importance of biological oscillators for hypothalamic–pituitary–adrenal activity and tissue glucocorticoid response: coordinating stress and neurobehavioural adaptation. J Neuroendocrinol.

[CR57] Sarkar A, Harty S, Lehto SM, Moeller AH, Dinan TG, Dunbar RIM (2018). The microbiome in psychology and cognitive neuroscience. Trends Cogn Sci.

[CR58] Scarantino A (2010). Insights and blindspots of the cognitivist theory of emotions. Br J Philos Sci.

[CR59] Scarantino A, de Sousa R (2018) Emotion. In: Zalta EN (ed) The Stanford encyclopedia of philosophy (Winter 2018 edition). https://plato.stanford.edu/archives/win2018/entries/emotion/. Accessed 20 June 2019

[CR60] Scherer KR (2009). The dynamic architecture of emotion: evidence for the component process model. Cogn Emot.

[CR61] Selye H (1936). A syndrome produced by diverse nocuous agents. Nature.

[CR62] Selye H (1950). Stress and the general adaptation syndrome. Br Med J.

[CR63] Shapiro LA (2014). The Routledge handbook of embodied cognition.

[CR64] Simonov PV (1986). The emotional brain: physiology, neuroanatomy, psychology, and emotion.

[CR65] Slavich GM, Irwin MR (2014). From stress to inflammation and major depressive disorder: a social signal transduction theory of depression. Psychol Bull.

[CR66] Spiga F, Walker JJ, Gupta R, Terry JR, Lightman SL (2015). Glucocorticoid dynamics: insights from mathematical, experimental and clinical studies. J Endocrinol.

[CR67] Spiga F, Zavala E, Walker JJ, Zhao Z, Terry JR, Lightman SL (2017). Dynamic responses of the adrenal steroidogenic regulatory network. PNAS.

[CR68] Stephan A (2017). Moods in layers. Philosophia.

[CR69] Stephan A, Walter S, Wilutzky W (2014). Emotions beyond brain and body. Philos Psychol.

[CR70] Sudo N, Chida Y, Aiba Y, Sonoda J, Oyama N, Yu X-N (2004). Postnatal microbial colonization programs the hypothalamic–pituitary–adrenal system for stress response in mice. J Physiol.

[CR71] Taylor VH (2019). The microbiome and mental health: hope or hype?. J Psychiatry Neurosci.

[CR72] Thagard P (2010). The brain and the meaning of life.

[CR73] Vanyukov MM, Moss HB, Plail JA, Blackson T, Mezzich AC, Tarter RE (1993). Antisocial symptoms in preadolescent boys and in their parents: associations with cortisol. Psychiatry Res.

[CR74] Vidal F, Ortega F (2017). Being brains: making the cerebral subject.

[CR75] Virkkunen M (1985). Urinary free cortisol secretion in habitually violent offenders. Acta Psychiatr Scand.

[CR76] Waite EJ, McKenna M, Kershaw Y, Walker JJ, Cho K, Piggins HD (2012). Ultradian corticosterone secretion is maintained in the absence of circadian cues. Eur J Neurosci.

[CR77] Walker JJ, Terry JR, Lightman SL (2010). Origin of ultradian pulsatility in the hypothalamic–pituitary–adrenal axis. Proc R Soc B Biol Sci.

[CR78] Wallace CJK, Milev R (2017). The effects of probiotics on depressive symptoms in humans: a systematic review. Ann Gen Psychiatry.

[CR79] Wehrenberg M, Prinz SM (2007). The anxious brain: the neurobiological basis of anxiety disorders and how to effectively treat them.

[CR80] Widmaier EP, Raff H, Strang KT (2014). Vander’s human physiology: the mechanisms of body function.

[CR81] Wilson RA (2001). Two views of realization. Philos Stud.

[CR82] Windle RJ, Wood SA, Lightman SL, Ingram CD (1998). The pulsatile characteristics of hypothalamo–pituitary–adrenal activity in female Lewis and Fischer 344 rats and its relationship to differential stress responses. Endocrinology.

[CR83] Windle RJ, Wood SA, Shanks N, Lightman SL, Ingram CD (1998). Ultradian rhythm of basal corticosterone release in the female rat: dynamic interaction with the response to acute stress. Endocrinology.

[CR84] Windle RJ, Wood SA, Kershaw YM, Lightman SL, Ingram CD, Harbuz MS (2001). Increased corticosterone pulse frequency during adjuvant-induced arthritis and its relationship to alterations in stress responsiveness. J Neuroendocrinol.

